# The modified cardiometabolic index as a risk factor for mortality after percutaneous coronary intervention in acute myocardial infarction

**DOI:** 10.1186/s12944-026-02935-0

**Published:** 2026-03-28

**Authors:** Tingting Xiao, Xiaoming Zhu, Shengjue Xiao, Jiandong Ding

**Affiliations:** 1 Department of Cardiology Zhongda Hospital, School of Medicine, Southeast University, 87 Dingjiaqiao, Nanjing, 210009 China; 2 Department of Cardiology, Xishan People’s Hospital of Wuxi City, Wuxi, 214000 China; 3 The Department of Geriatrics, The First Affiliated Hospital With Nanjing Medical University, Nanjing, Jiangsu 210029 China

**Keywords:** Acute myocardial infarction, All-cause mortality, Cardiovascular mortality, Percutaneous coronary intervention, Modified cardiometabolic index

## Abstract

**Objective:**

The cardiometabolic index (CMI) serves as an integrative measure of both obesity and dyslipidemia. This study aimed to evaluate whether an elevated modified cardiometabolic index (MCMI) is related to the risk of all-cause mortality (ACM) and cardiovascular mortality (CVM) after percutaneous coronary intervention (PCI) in patients with acute myocardial infarction (AMI).

**Methods:**

This large-scale retrospective cohort study utilized data from 2,688 participants with AMI undergoing PCI between June 2015 and June 2020 at Zhongda Hospital Southeast University and conducted follow-up. The MCMI, integrating high-density lipoprotein cholesterol, waist circumference, triglycerides, and fasting blood glucose, was used as a composite marker of cardiometabolic risk. After adjusting for potential confounding, restricted cubic splines (RCS), subgroup analyses, and Cox proportional hazards models were utilized to evaluate the associations of MCMI with ACM and CVM. Receiver operating characteristic (ROC) curve analysis was used to assess its diagnostic value.

**Results:**

In total, 2,688 participants with AMI were enrolled (mean age 54.47 ± 18.41 years, 50.9% male). The RCS plot revealed a positive linear association between MCMI and the risk of ACM and CVM in participants after AMI (all *P* for overall < 0.001). After adjusting for known confounders, compared with the lowest MCMI quartile, the hazard ratios (95% CIs) for ACM in the second, third, and fourth quartiles were 1.40 (1.07, 1.83), 1.53 (1.18, 1.99) and 1.55 (1.18, 2.03), respectively. The corresponding values for CVM were 1.32 (1.03, 2.06), 1.53 (1.15, 2.18) and 1.73 (1.21, 2.24). Finally, MCMI demonstrated better predictive ability for ACM and CVM in patients with AMI after PCI (AUC = 0.811; AUC = 0.828).

**Conclusions:**

In conclusion, this study demonstrates that among AMI patients, high MCMI was independently related to a greater risk of ACM and CVM. This finding establishes MCMI as a significant prognostic marker, which can aid in risk stratification and guide more aggressive secondary prevention strategies for high-risk individuals.

**Supplementary Information:**

The online version contains supplementary material available at 10.1186/s12944-026-02935-0.

## Background

Acute myocardial infarction (AMI) typically results from rupture or erosion of unstable atherosclerotic plaques, leading to persistent and complete coronary artery occlusion and subsequent acute ischemic necrosis. As the severe form of coronary artery disease (CAD), AMI is marked by substantial morbidity, high mortality, and poor prognosis [1]. Currently, the prevalence of AMI continues to rise, and it remains a significant global health concern. Percutaneous coronary intervention (PCI) has become the standard reperfusion strategy for AMI, effectively opening the occluded vessel and improving myocardial perfusion, which has led to its widespread adoption, which has significantly reduced AMI mortality [2]. Despite this, some patients still develop adverse cardiovascular events post-PCI, which are associated with substantially increased fatality rates [3]. Therefore, the early identification of high-risk patients for all-cause mortality (ACM) and cardiovascular mortality (CVM) after PCI is crucial for improving their prognosis.

Initially developed as a tool for identifying diabetes mellitus (DM), the Cardiometabolic Index (CMI) is defined as the product of the waist-to-height ratio (WHtR) and the triglyceride to high-density lipoprotein cholesterol ratio (TG/HDL-C) [4, 5]. In addition to diagnosing dysglycemia, CMI has been studied as an indicator to predict the risk and prognosis of cardiometabolic diseases. For instance, some scholars have investigated its relationship with the incidence of hypertension, suggesting value in early risk stratification [6]. Furthermore, CMI has been correlated with the presence of subclinical atherosclerosis, such as abdominal aortic calcification, and with the risk of atherosclerotic cardiovascular disease (ASCVD), particularly among individuals with DM [7, 8]. These findings highlight CMI's potential as a composite indicator of metabolic dysregulation relevant to diverse cardiovascular endpoints. To potentially enhance its predictive performance, a modified version (modified cardiometabolic index, MCMI) incorporating FBG has been introduced [9]. The key modification from the original CMI lies in the incorporation of fasting blood glucose (FBG) into the numerator, alongside triglycerides, to form the ratio TG × FBG/HDL-C. This adaptation aims to simultaneously capture two core components of cardiometabolic dysfunction-insulin resistance (reflected by FBG) and atherogenic dyslipidemia (reflected by TG/HDL-C)-thereby providing a more integrated measure of metabolic risk that may be particularly relevant in the acute post-infarction setting. Although the CMI and its modified form (MCMI) have demonstrated value in predicting risks across various metabolic disorders and general cardiovascular conditions, their prognostic utility specifically within the critical population of acute myocardial infarction particularly for long-term outcomes following revascularization-remains inadequately explored and lacks support from large-scale clinical evidence. To date, existing studies have been constrained by small cohorts, short follow-up, or a focus on surrogate endpoints rather than hard clinical outcomes such as mortality. Furthermore, no study has systematically evaluated whether MCMI predicts both ACM and CVM independently in the AMI population after accounting for established risk factors. Therefore, it was hypothesized that elevated MCMI at admission independently predicts long-term risk of ACM and CVM among AMI patients undergoing PCI. The present study represents the first large-scale cohort investigation to systematically evaluate the predictive value of MCMI for these endpoints in this specific population, potentially providing a simple, accessible tool for early risk stratification.

## Materials and methods

### Study population and design

This research collected data from the Electronic Medical Record system of patients treated at Zhongda Hospital, Southeast University. AMI patients confirmed by coronary angiography (CAG) and treated with PCI from June 2015 to June 2020 were included. Confidential patient data were removed before the analysis. As the study was based on anonymized clinical data, informed consent was therefore waived. The study was approved by the Institutional Review Board of Zhongda Hospital (No. 2020ZDSYLL051-P01).

### Inclusion and exclusion criteria

**Fig. 1:**
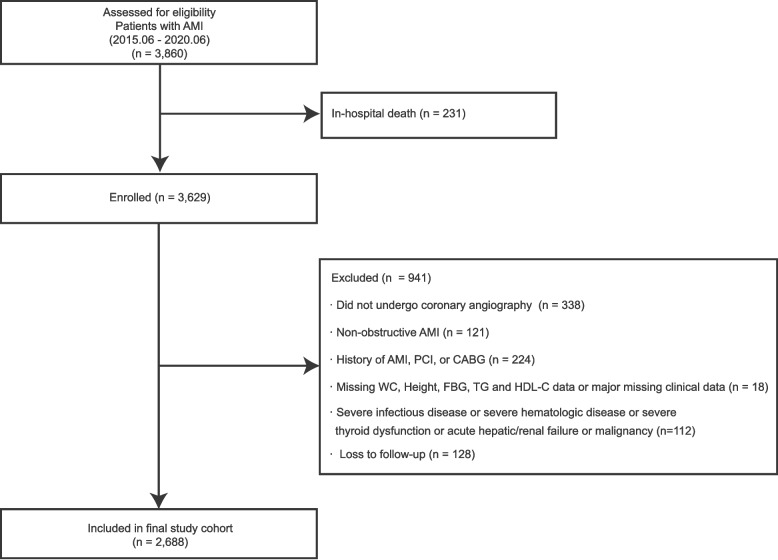
Study flow chart. Abbreviations: PCI, percutaneous transluminal coronary intervention

All participants were recruited from AMI patients who had received PCI and were admitted to Zhongda Hospital, Southeast University, between June 2015 and June 2020. Participants were included according to the following criteria: age ≥ 18 years, confirmed AMI diagnosis, and provision of informed consent for participation and follow-up. Key exclusion criteria were: absence of coronary angiography; prior AMI, PCI, or coronary artery bypass grafting; presence of severe concurrent conditions (including severe infections, hematological diseases, thyroid dysfunction, hepatorenal failure, or malignancies); death during the hospitalization; missing baseline data for height, HDL-C, waist circumference (WC), FBG or triglyceride (TG); and loss to follow-up. 3,860 consecutive patients treated with PCI and CAG for AMI between June 2015 and June 2020 were identified. After applying the pre-specified criteria, 1,172 patients (30.4%) were excluded due to incomplete information or meeting exclusion criteria, leaving 2,688 eligible patients for analysis (Fig. 1).

### Data collection

Comprehensive baseline information on all AMI patients was gathered for this analysis. This included key demographic variables—age, sex, and status regarding smoking and alcohol use. Furthermore, data on prior medical conditions were recorded, encompassing a history of hypertension, DM, heart failure, chronic kidney disease (CKD), and stroke. The serum biomarkers analyzed during the admission were also documented: alanine aminotransferase (ALT), hypersensitive troponin T, FBG, high-sensitivity C-reactive protein (Hs CRP), aspartate aminotransferase (AST), uric acid (UA), hemoglobin (Hb), blood urea nitrogen (BUN), creatine kinase MB (CKMB), platelet (Plt), white blood cell (WBC), creatine kinase (CK), neutrophil (Neu), lymphocyte (Lym), total cholesterol (TC), TG, red blood cell (RBC), estimated glomerular filtration rate (eGFR), N-terminal pro-brain natriuretic peptide, HDL-C, and serum creatinine (Scr). Additionally, physical examination indicators such as WC and height have also been included.

### Clinical endpoint and definitions

The study focused on ACM and CVM as the primary endpoints. ACM, defined as death from any cause, was determined through hospital medical records and follow-up contacts. CVM was defined as death primarily attributable to cardiac or vascular causes (AMI, heart failure, fatal arrhythmia, sudden cardiac death, cerebrovascular death, and other vascular causes). Participants were followed from the date of index PCI until death or study completion (median 40 months; interquartile range [IQR] 27–51 months), whichever came first. All suspected CVM events were adjudicated by blinded Clinical Endpoint Committee comprising two senior cardiologists, according to pre-specified standardized definitions adapted from the ACCF/AHA guidelines. The following definitions were applied: current smoking was defined as > 10 cigarettes/day for over one year; alcohol consumption was classified as sustained alcohol intake for > 5 years, or > 80 g/day in the last two weeks; a diagnosis of hypertension required blood pressure ≥ 140/90 mm Hg on ≥ 3 occasions. DM was defined as FBG > 7.0 mmol/L on ≥ 2 occasions or ongoing use of glucose-lowering medication [10]. The diagnosis of AMI was based on the Fourth Universal Definition of Myocardial Infarction [11].

### Definitions of MCMI and BMI

The MCMI is defined as: ln [TG (mg/dL) × FBG (mg/dL) / HDL-C (mg/dL)] × (WC (cm) / Height (cm)). This novel index modifies the original CMI, which is (TG/HDL-C) × (WC/Height), by incorporating fasting blood glucose into the numerator. This modification was designed to concurrently capture both dyslipidemia and glucose metabolism-related abnormalities, providing a more integrated assessment of cardiometabolic risk. Body mass index (BMI) was derived from weight (kg) / height squared (m^2^).

### Statistical analysis

Descriptive statistics were conducted. Continuous variables were reported as mean ± SD (independent Student's t-test); or as median with IQR (Mann–Whitney U test), depending on normality. Categorical variables were presented as n (%) and compared by Chi-square test. Based on MCMI values, patients were stratified into four quartiles (Q): Q1 (-0.616–0.568), Q2 (0.569–0.960), Q3 (0.961–1.413), and Q4 (1.414–4.641), with Q1 designated as the reference. Cox regression and restricted cubic splines (RCS) were used to assess the association between MCMI and mortality outcomes. Moreover, three multivariable models were conducted: Model 1 adjusted for sex and age; building upon Model 1, Model 2 adjusted for DM, smoking status, alcohol use, and hypertension additionally; and Model 3 further incorporated Hb, Plt, WBC, RBC, Lym, Neu, HbA1c, AST, ALT, total protein, TC, LDL, BUN, Scr, UA, eGFR, and Hs CRP. Additionally, receiver operating characteristic (ROC) curves were generated, and the area under the curve (AUC) with 95% confidence intervals (CIs) was calculated to assess the predictive performance of MCMI. The optimal cutoff value for the MCMI was determined using the Youden index. The post hoc power analysis, based on the observed effect size for primary outcomes (ACM and CVM), revealed an achieved power of 90%. This suggests that the study was adequately powered (above the conventional 80% threshold) to detect an effect of the observed magnitude. The sensitivity and specificity at this cutoff point were reported. To detect multicollinearity among candidate covariates, VIF values were calculated. Variables presenting VIF values above 5 were deemed to exhibit substantial collinearity and were consequently omitted from the multivariable regression models (Supplementary Table 1). Data were analyzed using SPSS (v22.0) and R (v4.2.3), with a significance level of 0.05.

## Results

### Baseline patient characteristics

As presented in Table 1, a total of 2,688 AMI participants were included. The cohort had a mean age of 54.47 ± 18.41 years and was 50.9% male. Nearly half of the patients had hypertension, and more than one quarter had DM and CKD, respectively. 60.2% presented with ST-segment elevation myocardial infarction (STEMI) and 39.8% with non-ST-segment elevation myocardial infarction (NSTEMI). In this cohort, revascularization was performed in 71.1% of participants within 12 h after symptom onset. The primary causes of in-hospital death were cardiogenic shock (42.1%), malignant arrhythmia (28.6%), reinfarction (15.2%), and other causes (14.1%). The data revealed substantial disparities across quartiles for the majority of variables. Notably, patients in higher MCMI quartiles (Q3 and Q4) were generally older and had more traditional cardiovascular risk factors (DM, hypertension, and smoking). Moreover, key metabolic parameters such as BMI, WC, FBG, HbA1c, and TG showed a clear gradient of increase across ascending MCMI quartiles (all *P* < 0.001). Crucially, ACM and CVM rates increased significantly with higher MCMI levels (*P* < 0.001).

**Table 1:** Characteristics of the study population based on MCMI quartiles

MCMI	Total (2,688)	Q1 (672)	Q2 (672)	Q3 (672)	Q4 (672)	*P*-value
Age, years	54.47 ± 18.41	48.89 ± 19.36	54.11 ± 18.79	56.81 ± 17.76	58.07 ± 16.25	< 0.001
Sex, n (%)						< 0.001
Male	1369 (50.9%)	270 (10.0%)	346 (12.9%)	367 (13.7%)	386 (14.4%)	
Female	1319 (49.1%)	402 (15.0%)	326 (12.1%)	305 (11.3%)	286 (10.6%)	
Hypertension, n (%)						< 0.001
No	1370 (51.0%)	442 (16.4%)	364 (13.5%)	306 (11.4%)	258 (9.6%)	
Yes	1318 (49.0%)	230 (8.6%)	308 (11.5%)	366 (13.6%)	414 (15.4%)	
DM, n (%)						< 0.001
No	1970 (73.3%)	626 (23.3%)	561 (20.9%)	468 (17.4%)	315 (11.7%)	
Yes	718 (26.7%)	46 (1.7%)	111 (4.1%)	204 (7.6%)	357 (13.3%)	
Smoker, n (%)						< 0.001
No	1411 (52.5%)	399 (14.8%)	368 (13.7%)	356 (13.2%)	288 (10.7%)	
Yes	1277 (47.5%)	273 (10.2%)	304 (11.3%)	316 (11.8%)	384 (14.3%)	
Alcohol user, n (%)						0.632
No	361 (13.4%)	94 (3.5%)	86 (3.2%)	94 (3.5%)	87 (3.2%)	
Yes	2327 (86.6%)	578 (21.5%)	586 (21.8%)	578 (21.5%)	585 (21.8%)	
HF, n (%)						< 0.001
No	2539 (94.5%)	649 (24.1%)	641 (23.8%)	631 (23.5%)	618 (23.0%)	
Yes	149 (5.5%)	23 (0.9%)	31 (1.2%)	41 (1.5%)	54 (2.0%)	
Stroke, n (%)						< 0.001
No	2532 (94.2%)	645 (24.0%)	631 (23.5%)	639 (23.8%)	617 (23.0%)	
Yes	156 (5.8%)	27 (1.0%)	41 (1.5%)	33 (1.2%)	55 (2.0%)	
CKD, n (%)						< 0.001
No	1996 (74.3%)	565 (21.0%)	528 (19.6%)	496 (18.5%)	407 (15.1%)	
Yes	692 (25.7%)	107 (4.0%)	144 (5.4%)	176 (6.5%)	265 (9.9%)	
AMI, n (%)						< 0.001
STEMI	1617 (60.2%)	419 (15.6%)	409 (15.2%)	402 (15.0%)	387 (14.4%)	
NSTEMI	1071 (39.8%)	253 (9.4%)	263 (9.8%)	270 (10.0%)	285 (10.6%)	
Revascularization within 12						
h of symptom onset, n (%)						< 0.001
No	776 (28.9%)	183 (6.8%)	192 (7.1%)	198 (7.4%)	203 (7.6%)	
Yes	1912 (71.1%)	489 (18.2%)	480 (17.9%)	474 (17.6%)	469 (17.4%)	
BMI, kg/m^2^	29.27 ± 6.98	24.95 ± 5.19	28.16 ± 6.08	30.25 ± 6.12	33.71 ± 7.28	< 0.001
WC, cm	100.65 ± 16.65	87.90 ± 12.86	98.04 ± 13.83	103.79 ± 13.27	112.88 ± 15.75	< 0.001
Height, cm	166.41 ± 10.00	165.94 ± 9.58	167.24 ± 10.05	166.34 ± 10.07	166.10 ± 10.27	0.078
Hb, g/dL	13.99 ± 1.59	13.62 ± 1.51	13.93 ± 1.60	14.12 ± 1.60	14.29 ± 1.57	< 0.001
Plt, 10^12/L	235.12 ± 65.42	230.82 ± 58.76	233.48 ± 71.81	239.68 ± 66.19	236.49 ± 64.08	0.074
WBC, 10^9/L	6.96 ± 2.53	6.00 ± 1.82	6.79 ± 2.98	7.34 ± 2.75	7.70 ± 2.06	< 0.001
RBC, 10^12/L	4.72 ± 0.54	4.61 ± 0.48	4.68 ± 0.56	4.77 ± 0.54	4.80 ± 0.56	< 0.001
Lym, 10^9/L	1.90(1.50–2.40)	1.80(1.40–2.20)	1.90(1.50–2.30)	2.00(1.60–2.50)	2.10(1.70–2.60)	< 0.001
Neu, 10^9/L	4.08 ± 1.69	3.43 ± 1.49	3.97 ± 1.73	4.30 ± 1.63	4.63 ± 1.65	< 0.001
FBG, mmol/L	6.40 ± 2.25	5.44 ± 0.61	5.78 ± 1.09	6.35 ± 1.68	8.04 ± 3.45	< 0.001
HbA1c	5.93 ± 1.24	5.43 ± 0.50	5.64 ± 0.78	5.98 ± 1.11	6.69 ± 1.78	< 0.001
AST, U/L	21.00(18.00–27.00)	21.00(17.00–25.00)	22.00(18.00–26.00)	21.00(18.00–27.00)	23.00(18.00–28.25)	0.434
ALT, U/L	14.00 (19.00–27.00)	17.00(13.00–22.00)	19.00(14.00–25.00)	20.00(15.00–29.00)	24.00(17.00–33.00)	0.011
Albumin, g/dL	41.28 ± 3.72	41.91 ± 3.52	41.11 ± 3.88	41.36 ± 3.59	40.75 ± 3.77	< 0.001
TC, mmol/L	4.87 ± 1.09	4.70 ± 0.96	4.82 ± 1.06	4.87 ± 1.07	5.10 ± 1.21	< 0.001
TG, mmol/L	1.10(0.75–1.63)	0.59(0.47–0.72)	0.94(0.78–1.14)	1.33(1.10–1.62)	2.06(1.58–2.74)	< 0.001
HDL-C, mmol/L	1.40 ± 0.43	1.81 ± 0.44	1.47 ± 0.32	1.25 ± 0.26	1.06 ± 0.24	< 0.001
LDL, mmol/L	2.86 ± 0.96	2.60 ± 0.82	2.90 ± 0.91	2.98 ± 0.95	2.96 ± 1.08	< 0.001
BUN, mmol/L	5.53 ± 2.64	5.06 ± 2.16	5.39 ± 2.23	5.64 ± 2.76	6.04 ± 3.18	< 0.001
Scr, µmol/L	76.02 (61.88–90.17)	72.49(59.23–86.63)	76.02(62.76–89.28)	76.02 (61.90–93.70)	78.68 (61.88–95.47)	0.085
UA, µmol/L	332.11 ± 90.71	290.74 ± 73.90	322.09 ± 84.29	347.96 ± 90.19	367.67 ± 94.42	< 0.001
eGFR, mL/min/1.73m^2^	90.55 ± 26.23	97.46 ± 25.16	91.19 ± 25.58	88.02 ± 25.43	85.51 ± 27.20	< 0.001
Hs CRP, mg/dL	3.48 ± 0.14	2.15 ± 0.26	3.42 ± 0.21	3.84 ± 0.21	4.75 ± 0.28	< 0.001
CK, U/L	590.46 ± 10.13	360.68 ± 9.64	575 ± 10.26	645 ± 10.73	782 ± 9.69	< 0.001
CKMB, ng/mL	86.16 ± 4.56	53.16 ± 4.12	84 ± 4.68	94 ± 4.02	114 ± 4.85	< 0.001
Hs TnT, ng/L	712.78 ± 18.28	440 ± 18.49	698 ± 17.94	782 ± 18.16	931 ± 17.46	< 0.001
NT-proBNP, pg/mL	742.56 ± 24.64	458 ± 24.89	728 ± 25.16	815 ± 24.73	969 ± 27.46	< 0.001
All-cause mortality, n (%)						< 0.001
No	2115 (78.7%)	585 (21.8%)	534 (19.9%)	508 (18.9%)	488 (18.2%)	
Yes	573 (21.3%)	87 (3.2%)	138 (5.1%)	164 (6.1%)	184 (6.8%)	
CVD Mortality, n (%)						< 0.001
No	2491 (92.7%)	640 (23.8%)	622 (23.1%)	620 (23.1%)	609 (22.7%)	
Yes	197 (7.3%)	32 (1.2%)	50 (1.9%)	52 (1.9%)	63 (2.3%)	

*Abbreviations Q1* 1.00–10.50 ng/mL, *Q2* 10.51–15.90 ng/mL, *Q3* 15.91–24.90 ng/mL, *Q4* 24.91–614.00 ng/mL, *DM* Diabetes mellitus, *HF* Heart failure, *CKD* Chronic kidney disease, *h* hours, *BMI* Body mass index, *WC* Waist circumference, *FBG* Fasting blood glucose; *ALT* Alanine aminotransferase, *AST* Aspartate aminotransferase, *Hb* Hemoglobin, *Plt* Platelet, *WBC* White blood cell, *RBC* Red blood cell, *Lym* Lymphocyte, *Neu* Neutrophil, *TC* Total cholesterol, *TG* Triglyceride, *HDL-C* High density lipoprotein- cholesterol, *BUN* Blood urea nitrogen, *UA* Uric acid, *Scr* Serum creatinine, *eGFR* estimated glomerular filtration rate, *Hs CRP* High-sensitivity C-reactive protein, *CK* Creatine kinase, *CKMB* Creatine kinase MB, *hsTn T* Hypersensitive troponin T, *NT-proBNP* N-terminal pro-brain natriuretic peptide, *CVD* Cardiovascular disease

### Association of MCMI with all-cause and cardiovascular mortality

**Fig. 2:**
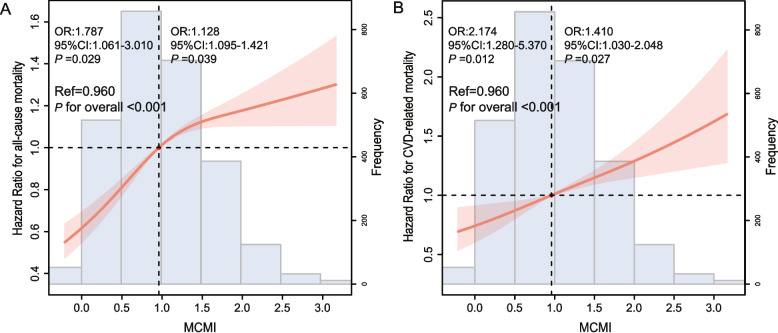
RCS plots of associations between MCMI and in AMI patients after PCI. **A** All-cause mortality; **B** Cardiovascular mortality. Abbreviations: RCS, restricted cubic spline; MCMI, Modified cardiometabolic index; PCI, percutaneous transluminal coronary intervention; AMI, Acute myocardial infarction

**Fig. 3:**
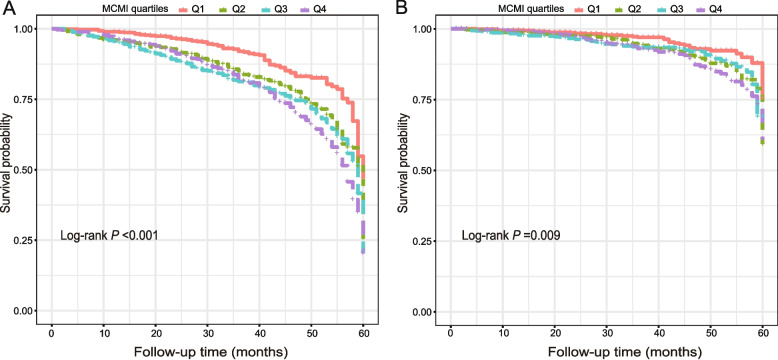
Kaplan–Meier survival curve for all-cause and cardiovascular mortality in AMI patients after PCI. **A** All-cause mortality; **B** Cardiovascular mortality. Abbreviation: PCI, percutaneous transluminal coronary intervention; AMI, Acute myocardial infarction

The RCS model demonstrated that MCMI was positively and linearly related to the risks of ACM and CVM in patients (*P* for overall < 0.001; Fig. 2). After multivariable adjustment (Model 3), with the lowest MCMI quartile (Q1) as the reference, the hazard ratios (HRs) and 95% CIs for ACM across the Q2, Q3, and Q4 were 1.40 (1.07–1.83), 1.53 (1.18–1.99), and 1.55 (1.18–2.03), respectively. The corresponding HRs (95% CIs) for CVM were 1.32 (1.03–2.06), 1.53 (1.15–2.18), and 1.73 (1.21–2.24) (Tables 2 and 3). Kaplan–Meier analysis according to MCMI quartiles showed a significant gradient of risk for both ACM (Fig. 3A) and CVM (Fig. 3B). Notably, individuals in Q4 experienced the poorest survival outcomes (Log-rank *P* < 0.001 for ACM; *P* = 0.009 for CVM).

**Table 2:** Adjusted HR for associations between MCMI and all-cause mortality

	Model 1		Model 2		Model 3	
	HR (95%CI)	*P* for trend	HR (95%CI)	*P* for trend	HR (95%CI)	*P* for trend
MCMI		< 0.001		< 0.001		0.002
Q1	1.00		1.00		1.00	
Q2	1.43 (1.09, 1.86) **		1.43 (1.09, 1.87) **		1.40 (1.07, 1.83) **	
Q3	1.46 (1.12, 1.89) **		1.52 (1.14, 1.97) **		1.53 (1.18, 1.99) **	
Q4	1.59 (1.24, 2.05) ***		1.68 (1.29, 2.20) ***		1.55 (1.18, 2.03) **	

Model 1: age and sex

Model 2: model 1 variables plus the complication of hypertension, and diabetes mellitus, smoker, and alcohol user

Model 3 was adjusted for model 2 variables plus the complication of chronic heart failure, stroke, and chronic kidney disease, hemoglobin, alanine aminotransferase, aspartate aminotransferase, platelet; white blood cell; red blood cell; lymphocyte; neutrophil, total protein, low-density lipoprotein cholesterol, total cholesterol, blood urea nitrogen, uric acid, serum creatinine, estimated glomerular filtration rate and high-sensitivity C-reactive protein

*Abbreviations MCMI* Modified cardiometabolic index, MCMI (Q1, -0.616–0.568, *Q2*, 0.569–0.960, *Q3* 0.961–1.413, *Q4* 1.414–4.641), *HR* Hazard ratio, *CI* Confidence interval

^**^P < 0.01; ***P < 0.001

**Table 3:** Adjusted HR for associations between MCMI and cardiovascular mortality

	Model 1		Model 2		Model 3	
	HR (95%CI)	*P* for trend	HR (95%CI)	*P* for trend	HR (95%CI)	*P* for trend
MCMI		< 0.001		0.051		0.169
Q1	1.00		1.00		1.00	
Q2	1.37 (1.08, 1.97) **		1.35 (1.06, 2.12) **		1.32 (1.03, 2.06) **	
Q3	1.69 (1.18, 2.17) ***		1.61 (1.17, 2.20) ***		1.53 (1.15, 2.18) ***	
Q4	1.89 (1.27, 2.79) ***		1.75 (1.24, 2.60) ***		1.73 (1.21, 2.24) ***	

Model 1: age and sex

Model 2: model 1 variables plus the complication of hypertension, and diabetes mellitus, smoker, and alcohol user

Model 3 was adjusted for model 2 variables plus the complication of chronic heart failure, stroke, and chronic kidney disease, hemoglobin, alanine aminotransferase, aspartate aminotransferase, platelet; white blood cell; red blood cell; lymphocyte; neutrophil, total protein, low-density lipoprotein cholesterol, total cholesterol, blood urea nitrogen, uric acid, serum creatinine, estimated glomerular filtration rate and high-sensitivity C-reactive protein

*Abbreviations MCMI* Modified cardiometabolic index, MCMI (Q1, -0.616–0.568, *Q2* 0.569–0.960, *Q3* 0.961–1.413, *Q4* 1.414–4.641). *HR* Hazard ratio, *CI* Confidence interval

^**^P < 0.01; ***P < 0.001

### Subgroup analysis

Subgroup analyses based on age, sex, DM and hypertension were further performed (Supplementary Figs. 1 and 2). A linear positive correlation of MCMI with ACM risk was found among AMI patients after PCI who were aged < 60 years, male or female, had hypertension, with or without DM (Supplementary Fig. 1). Additionally, there was linear positive correlation between MCMI and CVM risk among patients who were aged < 60 years, male, with hypertension, with or without DM (Supplementary Fig. 2).

### Diagnostic value of MCMI

**Fig. 4:**
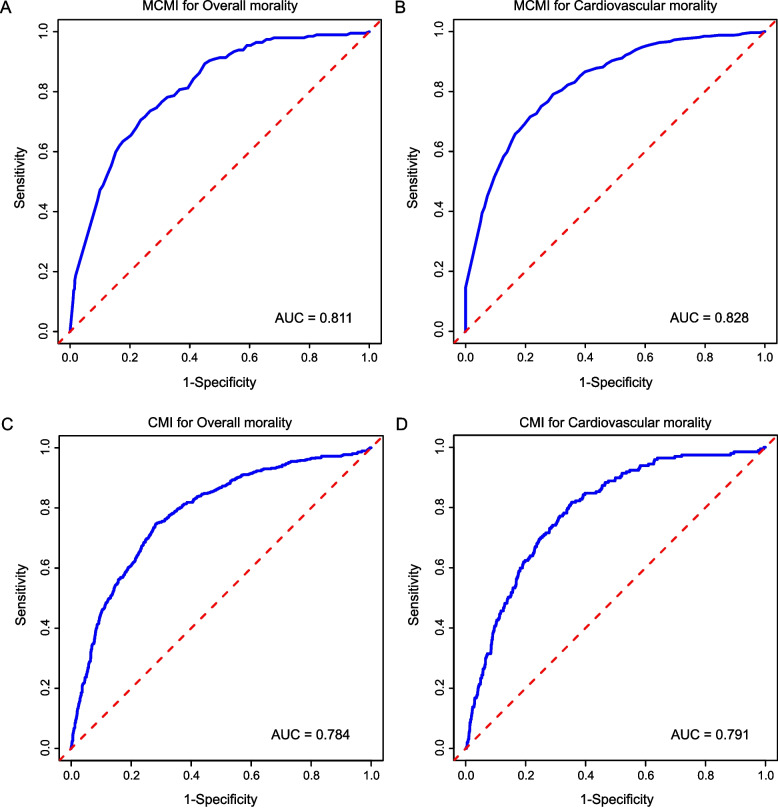
ROC curve for predicting the risk of all-cause and cardiovascular mortality in patients with AMI after PCI. **A** The AUC of MCMI for all-cause mortality = 0.811, with a sensitivity of 70.6% and a specificity of 76.4%. **B** The AUC of MCMI for cardiovascular mortality = 0.828, with a sensitivity of 71.6% and a specificity of 78.4%. **C** The AUC of CMI for all-cause mortality = 0.784, with a sensitivity of 68.9% and a specificity of 73.4%. **D** The AUC of MCMI for cardiovascular mortality = 0.791, with a sensitivity of 69.1% and a specificity of 74.7%. Note: The x axis represents the false-positive rate, while the y axis shows the true-positive rate. Abbreviations: ROC, Receiver operating characteristic; AUC, the area under ROC curve; PCI, percutaneous transluminal coronary intervention; AMI, Acute myocardial infarction

The MCMI showed strong predictive performance for both ACM and CVM outcomes. For ACM, the ROC curve yielded an AUC of 0.811, with 70.6% sensitivity and 76.4% specificity (Fig. 4A). For CVM, the AUC was 0.828, with 71.6% sensitivity and 78.4% specificity (Fig. 4B). Additionally, as presented in Figs. 4C and D, the CMI demonstrated statistically significant inferior predictive performance for ACM and CVM compared with the novel MCMI. The predictive accuracy of CMI was assessed by ROC curve. The AUC for overall mortality was 0.784, and for cardiovascular disease-related mortality, it was 0.791.

## Discussion

This research represents one of the first and largest cohort studies to elucidate the positive relationship between the MCMI and the risks of ACM and CVM among AMI patients undergoing PCI. This finding not only confirms the independent prognostic value of MCMI in this high-risk population but also provides a fresh perspective on the role of cardiometabolic dysfunction in the prognosis of CAD. The findings are consistent with previous studies linking cardiometabolic risk factors to cardiovascular outcomes but take a step further. Based on the ROC analysis (Youden index), an MCMI score of 2.14 was identified as the optimal cutoff for distinguishing high-risk patients in the cohort. Patients with an MCMI ≥ 2.14 had a 3.2-fold higher risk of mortality (ACM and CVM) than those below this threshold. Traditional risk stratification has largely relied on conventional risk factors or simple metabolic indices, such as FBG and triglyceride-glucose (TyG) index [12–14]. The innovation of the MCMI, however, is the integration of plasma triglyceride levels (a marker of dyslipidemia) and fasting glucose (a reflection of insulin resistance) into a single composite index [15, 16]. This integration may more comprehensively capture the pathophysiological state that drives atherosclerotic progression and plaque instability. Furthermore, the prognostic value of MCMI was found to be independent of traditional risk factors, including DM, suggesting that incorporating MCMI into conventional risk models may obviously enhance the predictive accuracy for post-PCI patients. The underlying mechanisms linking MCMI to adverse outcomes are likely multifactorial. Firstly, insulin resistance promotes endothelial dysfunction, exacerbates inflammatory responses, and impairs the fibrinolytic system, thus accelerating atherosclerosis and causing higher risk of thrombus formation [17]. Secondly, hypertriglyceridemia is frequently associated with elevated levels of atherogenic lipoprotein remnants, which more readily infiltrate the vascular wall, driving plaque formation and progression [18]. Consequently, the "glucolipotoxic" environment represented by MCMI may act synergistically, collectively fostering a more aggressive form of CAD and worse clinical outcomes. The impact of this systemic metabolic derangement persists even after revascularization. From a clinical practice perspective, these findings carry significant implications. The MCMI, being a simple and cost-effective calculable index, can be readily integrated into routine clinical workflows. Patients exhibiting an elevated MCMI following AMI and PCI may thus be identified as a "high-risk subgroup" warranting more intensive follow-up and aggressive management. Such management could include, but is not limited to, intensified lipid-lowering therapy (e.g., PCSK9 inhibitors), stricter glycemic control, and comprehensive cardiac rehabilitation with lifestyle interventions [19]. Furthermore, the association between elevated MCMI and higher mortality risk remained statistically significant and independent after extensive adjustment for a range of potential confounders. Specifically, Model 3 included demographics, lifestyle factors, clinical comorbidities, and a comprehensive set of laboratory biomarkers (including lipid profiles, HbA1c, renal function, and inflammatory markers), and MCMI remained a strong predictor of both ACM and CVM. This indicates that the prognostic information captured by MCMI is not redundant with these established traditional and metabolic risk factors. The predictive performance of MCMI in this cohort was robust, with an AUC of 0.811 for ACM and 0.828 for CVM. This aligns with the reported performance of other emerging composite metabolic indices for risk stratification in AMI. For example, the TyG index and the estimated glucose disposal rate (eGDR) have demonstrated similar prognostic value in contemporary studies [20–23]. This consistency underscores the potential of indices that integratively reflect glucolipid metabolism as robust tools for identifying high-risk patients post-AMI. A distinctive aspect of MCMI, however, is its explicit incorporation of central adiposity (via waist-to-height ratio) alongside glycemic and lipid parameters. This design may allow MCMI to capture a broader "metabolic triad" of obesity, insulin resistance, and dyslipidemia, potentially offering a more holistic risk profile than indices focused predominantly on glucose and lipid metabolism. In addition to mortality risk, bleeding complications following PCI represent a critical determinant of long-term prognosis in AMI patients. Major bleeding events are independently associated with higher mortality and may indirectly increase ischemic risk through premature discontinuation or modification of antithrombotic therapy. Recently, several bleeding risk prediction tools have been developed to facilitate individualized risk stratification. Notably, the recently proposed PRECISE-HBR score demonstrated good discriminative ability for predicting major bleeding events after PCI and outperformed traditional bleeding risk models in external validation cohorts [24]. Incorporating such bleeding risk assessment tools into post-PCI management may help optimize the balance between ischemic protection and bleeding risk. Although this research focuses on the relation between MCMI and mortality outcomes, future studies may explore whether integrating metabolic risk indices with established bleeding risk scores could provide a more comprehensive prognostic framework for patients with AMI undergoing PCI. Future research should focus on establishing validated risk cut-off values for MCMI and confirming its generalizability across diverse populations through large-scale, multi-center cohort studies.

## Strengths and limitations

Several notable strengths of this study warrant consideration. First, based on current literature, this is one of the first and largest cohort studies to systematically investigate the relation between the MCMI and the risks of ACM and CVM among AMI patients undergoing PCI. Statistical power and generalizability were enhanced by the large sample size and well-characterized cohort. Second, the MCMI is a novel and readily applicable prognostic tool. As a composite metric derived from routine laboratory parameters (FBG, TG, HDL-C, WC, and height), it is both cost-effective and easily implementable in diverse clinical settings. Unlike complex biomarkers or imaging-based risk scores, MCMI can be calculated rapidly without additional cost or specialized equipment, facilitating its potential for immediate translation into clinical practice for risk stratification in post-PCI patients. Third, the study employed rigorous statistical methodologies, including RCS to explore dose–response relationships, comprehensive multivariable adjustment, and subgroup analyses to assess consistency across populations. These approaches strengthen the robustness and reliability of the findings. Fourth, the integration of central adiposity alongside glycemic and lipid parameters distinguishes MCMI from other metabolic indices, offering a more holistic assessment of cardiometabolic risk. This may enhance its utility in identifying high-risk individuals for targeted prevention. Collectively, these strengths underscore the clinical and scientific value of MCMI as a simple, accessible, and powerful tool for improving risk stratification and guiding secondary prevention. Nevertheless, some limitations exist in this study. Firstly, due to its single-center observational design, the findings may be constrained by selection bias and the limited sample size, which could affect their generalizability to broader populations. Secondly, despite extensive adjustment for known clinical confounders, the possibility of residual confounding cannot be ruled out. Unmeasured factors (e.g., psychosocial stress, dietary habits, and medication adherence) may have influenced both the MCMI and the mortality outcomes, potentially biasing the estimates. Thirdly, the calculation of MCMI relies on single-point measurements of fasting blood glucose and triglycerides, which are subject to biological variability. The lack of repeated measurements to calculate a time-averaged MCMI value might not accurately reflect the participants' long-term cardiometabolic status, potentially leading to misclassification. Fourthly, a notable proportion of the initial cohort (1,172 patients, 30.36%) was excluded due to missing data or failure to meet inclusion criteria, which may introduce selection bias. This exclusion potentially affects the representativeness of our study population, as those with incomplete records or more severe comorbidities might differ systematically from the analyzed sample. However, it is important to note that such exclusions are common in retrospective cohort studies relying on electronic health records, and consistent, pre-specified criteria were applied to minimize arbitrary selection. Despite this, the potential for bias cannot be entirely ruled out, and future research with more complete data collection is warranted to verify these results in a more representative cohort. Fifthly, the in-hospital mortality rate observed in our cohort (5.9%) was higher than some reported national averages. This likely reflects the nature of our institution as a major tertiary referral center, which admits a higher proportion of patients with severe, complex, or high-risk AMI. Consequently, our study population may not be fully representative of all AMI patients treated with PCI in community or non-referral hospital settings. This inherent selection bias, while providing valuable insights into a high-risk subgroup, may limit the direct generalizability of our absolute risk estimates (such as mortality rates) to the broader AMI population. However, given that the primary aim was to investigate the MCMI-mortality risk relationship, the internal validity of this association is likely robust within this well-characterized cohort. Sixthly, while a significant independent relation exists between MCMI and mortality, formal evaluation of its incremental predictive value over established clinical models was not performed. Future studies should assess whether adding MCMI to conventional risk scores (e.g., GRACE score) significantly improves risk discrimination or reclassification in post-PCI AMI patients. Such analyses, preferably in large, prospective, multi-center cohorts with pre-specified validation sets, are necessary to fully establish the clinical utility of implementing MCMI in routine practice. Additionally, a key limitation is that FBG and TG are modified by pharmacotherapy. Thus, an elevated MCMI in treated patients may indicate residual risk or treatment resistance, not merely baseline risk. The lack of detailed medication data precluded adjustment for treatment intensity (a proxy for disease severity), potentially introducing residual confounding. This obscures whether MCMI’s prognostic value is intrinsic or mediated by treatment intensity. Future studies with detailed, longitudinal medication data are needed. Finally, the cohort consisted exclusively of AMI patients undergoing PCI. Therefore, these findings may not be generalizable to people with other forms of coronary artery disease, those managed medically or with coronary artery bypass grafting, or to healthier community-dwelling populations. The generalizability of the proposed MCMI cut-off values to other ethnicities requires external validation.

## Conclusion

This study provides robust evidence that the MCMI is an independent predictor of long-term ACM and CVM in patients with AMI after PCI. Higher MCMI levels were significantly associated with increased risks of ACM and CVM, underscoring the prognostic importance of cardiometabolic dysfunction in this population. As a simple, cost-effective index derived from routine clinical parameters (FBG, TG, HDL-C, WC, and height), MCMI enhances risk stratification beyond conventional risk factors. In clinical practice, MCMI can be readily calculated to identify high-risk patients who may benefit from more intensive follow-up and targeted interventions, including intensified lipid-lowering therapy, stricter glycemic control, and comprehensive cardiac rehabilitation. Early identification of these individuals enables personalized secondary prevention strategies, ultimately aiming to improve survival and cardiovascular outcomes. Future multicenter prospective studies are warranted to validate optimal MCMI cutoffs and evaluate its generalizability across diverse populations.

## Supplementary Information

Supplementary Material 1. Supplementary Figure 1. The RCS curve of the association of MCMI with all-cause mortality risk based on (A) age, (B) sex, (C) hypertension and (D) diabetes mellitus in AMI patients after PCI.

Supplementary Material 2. Supplementary Figure 2. The RCS curve of the association of MCMI with cardiovascular mortality risk based on (A) age, (B) sex, (C) hypertension and (D) diabetes mellitus in AMI patients after PCI.

Supplementary Material 3. Supplementary Table 1. Multicollinearity tests based on Model 3.

## Data Availability

Data supporting this study are available from the corresponding author upon reasonable request.
