# Supplementary figures and images for: The modified cardiometabolic index as a risk factor for mortality after percutaneous coronary intervention in acute myocardial infarction
Source: Lipids Health Dis. 2026 Mar 28;25:125. doi: 10.1186/s12944-026-02935-0 (PMC13151322; doi:10.1186/s12944-026-02935-0)

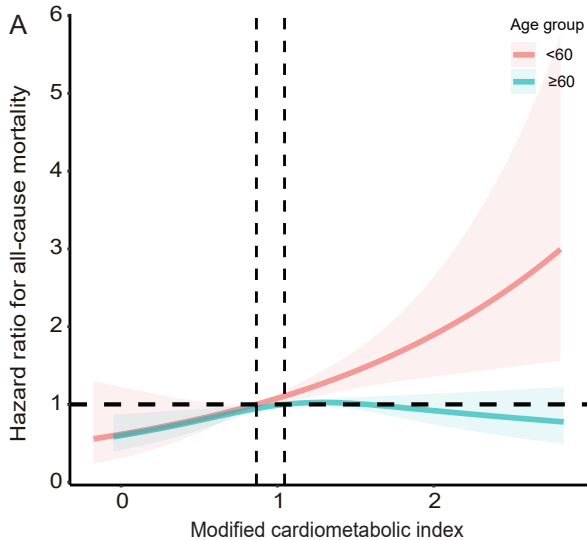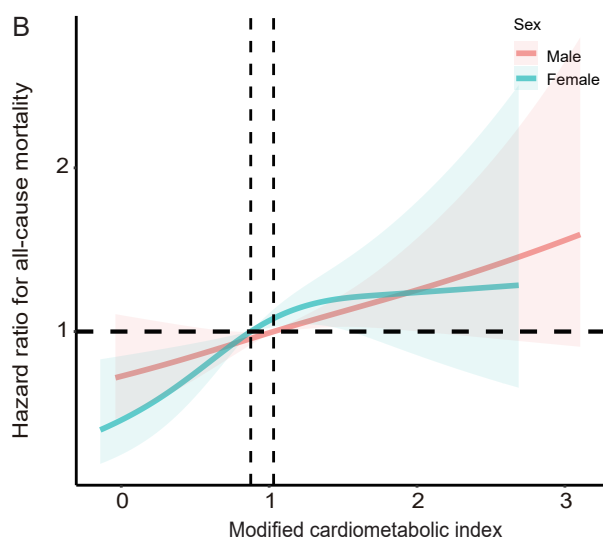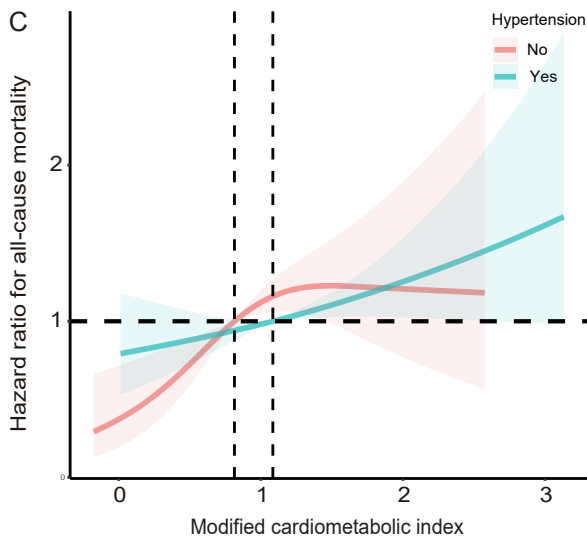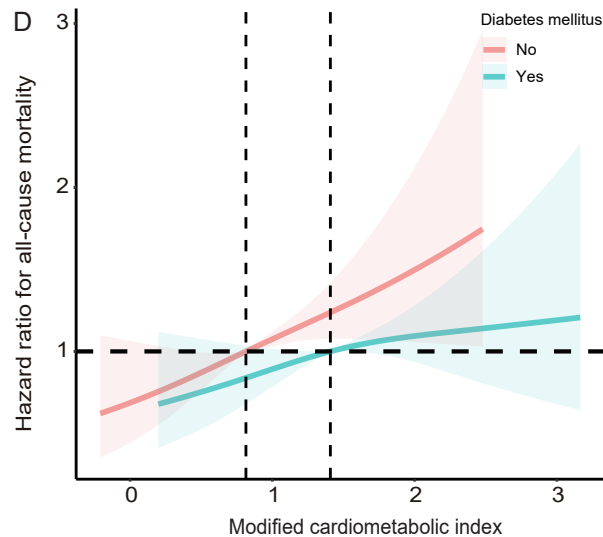

Supplement: Supplementary file 1 — Supplementary Material 1. Supplementary Figure 1. The RCS curve of the association of MCMI with all-cause mortality risk based on (A) age, (B) sex, (C) hypertension and (D) diabetes mellitus in AMI patients after PCI. [file 12944_2026_2935_MOESM1_ESM.pdf]

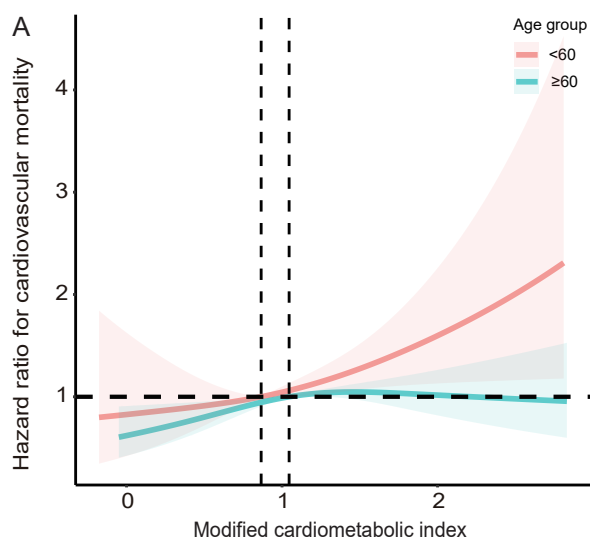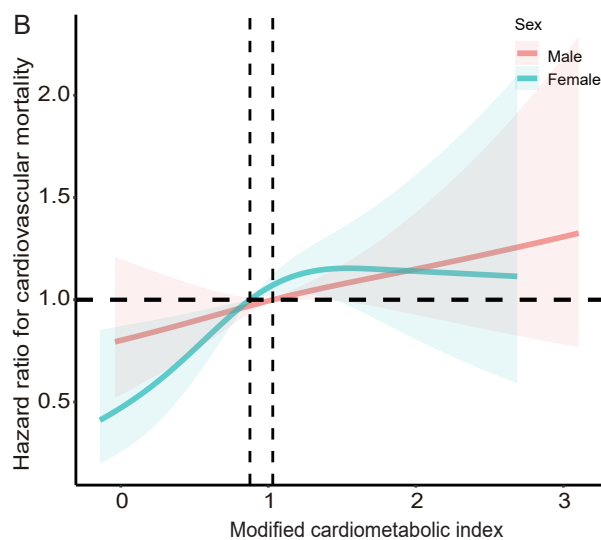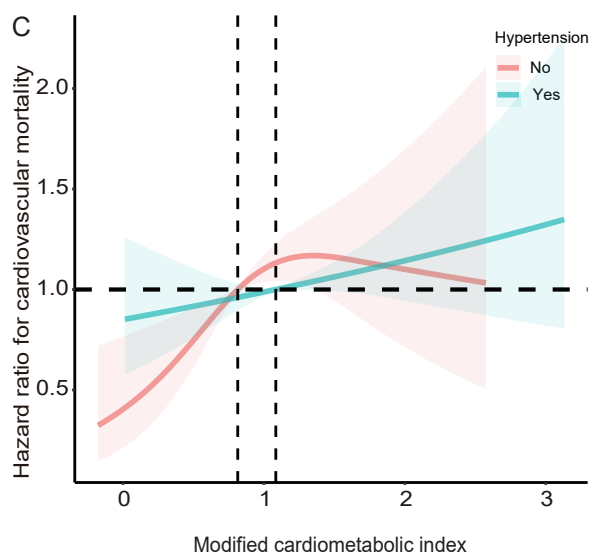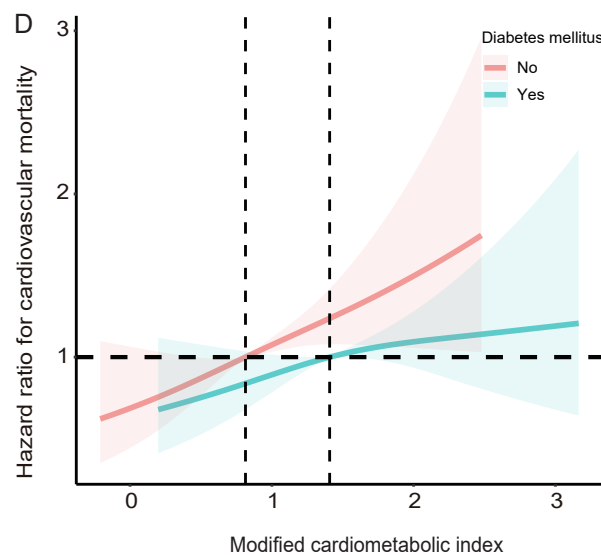

Supplement: Supplementary file 2 — Supplementary Material 2. Supplementary Figure 2. The RCS curve of the association of MCMI with cardiovascular mortality risk based on (A) age, (B) sex, (C) hypertension and (D) diabetes mellitus in AMI patients after PCI. [file 12944_2026_2935_MOESM2_ESM.pdf]
