# Supplementary material for: The modified cardiometabolic index as a risk factor for mortality after percutaneous coronary intervention in acute myocardial infarction
Source: Lipids Health Dis. 2026 Mar 28;25:125. doi: 10.1186/s12944-026-02935-0 (PMC13151322; doi:10.1186/s12944-026-02935-0)
Supplement: Supplementary file 3 — Supplementary Material 3. Supplementary Table 1. Multicollinearity tests based on Model 3. [file 12944_2026_2935_MOESM3_ESM.docx]

| **Supplementary Table 1.** Multicollinearity tests based on Model 3. | |
| --- | --- |
| Variable | VIF |
| Age, years | 1.626 |
| Sex, % | 1.613 |
| Hypertension, % | 1.100 |
| DM, % | 1.356 |
| Smoker, % | 1.131 |
| Drinker, % | 1.119 |
| HF, % | 1.063 |
| Stroke, % | 1.038 |
| CKD, % | 1.252 |
| BMI, kg/m^2^ | 2.867 |
| WC, cm | 3.026 |
| Height, cm | 1.629 |
| Hb, g/dL | 1.797 |
| Plt, 10^12/L | 1.131 |
| WBC, 10^9 | 1.372 |
| RBC, 10^9 | 1.693 |
| Lym, 10^9/L | 2.758 |
| Neu, 10^9/L | 2.840 |
| FBG, mmol/L | 1.682 |
| HbA1c | 1.778 |
| AST, U/L | 2.854 |
| ALT, U/L | 2.860 |
| Albumin, g/dL | 1.130 |
| TC, mmol/L | 2.384 |
| TG, mmol/L | 2.851 |
| HDL-C, mmol/L | 2.625 |
| LDL, mmol/L | 2.994 |
| BUN, mmol/L | 1.500 |
| Scr, µmol/L | 1.566 |
| UA, µmol/L | 1.289 |
| eGFR, mL/min/1.73m² | 2.131 |
| Hs CRP, mg/dL | 1.117 |
| CK, U/L | 2.256 |
| CKMB, ng/mL | 1.486 |
| Hs TnT, ng/L | 1.649 |
| NT-proBNP, pg/mL | 1.879 |

Abbreviations: VIF, variance inflation factor; DM, diabetes mellitus; HF, heart failure; CKD, chronic kidney disease; BMI, body mass index; WC, Waist circumference; FBG, fast glucose; Alt, alanine aminotransferase; Ast, aspartate aminotransferase; Hb, hemoglobin; Plt, platelet; WBC, White blood cell; RBC, Red blood cell; Lym, lymphocyte; Neu, Neutrophil; TC, total cholesterol; TG, triglyceride; HDL-C, high density lipoprotein- cholesterol; BUN, blood urea nitrogen; UA, uric acid; Scr, serum creatinine; eGFR, estimated glomerular filtration rate; Hs CRP, High-sensitivity C-reactive protein; CK, creatine kinase; CKMB, creatine kinase MB; hsTnT, hypersensitive troponin T; NT-proBNP, N-terminal pro-brain natriuretic peptide;
